# Management of general anesthesia in a child with Miller–Dieker syndrome: a case report

**DOI:** 10.1186/s40981-015-0017-2

**Published:** 2015-09-18

**Authors:** Chiaki Wakiguchi, Kohei Godai, Keika Mukaihara, Tetsuya Ohnou, Tamotsu Kuniyoshi, Mina Masuda, Yuichi Kanmura

**Affiliations:** Department of Anesthesiology and Critical Care Medicine, Graduate School of Medical and Dental Sciences, Kagoshima University, 8-35-1 Sakuragaoka, Kagoshima, 890-8520 Japan

**Keywords:** Miller–Dieker syndrome, Lissencephaly, Bispectral Index

## Abstract

Miller–Dieker syndrome (MDS) is a rare disorder characterized by type I lissencephaly and a distinctive facial appearance that may include prominent forehead, bitemporal hollowing, and micrognathia. MDS is associated with epilepsy. We here report an 18-month-old girl with MDS who required general anesthesia. The child had an extremely low Bispectral Index (BIS) value prior to undergoing general anesthesia. Her perioperative course was uneventful. This case highlights some of the important anesthetic concerns in patients with MDS, which include potentially difficult airways and extremely low BIS values.

## Background

Miller–Dieker syndrome (MDS) is a rare disorder that is characterized by type I lissencephaly (smooth brain), a distinctive facial appearance, and often other abnormalities [1]. The characteristic facial appearance includes a prominent forehead, bitemporal hollowing, short nose with upturned nares, prominent upper lip, and micrognathia. MDS is associated with the epilepsy like West syndrome and severe mental deficiency.

To the best of our knowledge, only one case describing administration of anesthesia to a patient with MDS has been reported. That case report, however, focuses only on the electroencephalographic findings during general anesthesia [2]. The detailed anesthetic management of patients with MDS has not previously been reported. We here report, for the first time, the anesthetic management of a patient with MDS.

## Case presentation

**Fig. 1:**
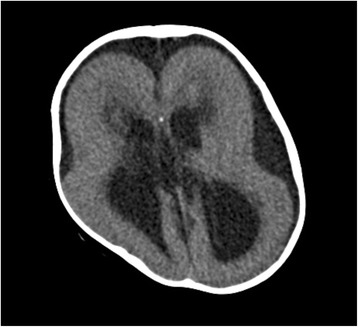
Head computed tomography (CT). Head CT showing type I lissencephaly with midline calcification

An 18-month-old girl weighing 6.9 kg was scheduled for elective laryngotracheal separation and percutaneous gastrostomy under general anesthesia. An antenatal ultrasound scan at 30 weeks had shown retardation of fetal growth and oligohydramnios. Delivery had been by cesarean section at 38 weeks gestation. She had a birth weight of 2064 g, and Apgar scores of 3 and 8 at 1 and 5 min, respectively. Head ultrasound had shown dilated bilateral ventricles and head computed tomography type I lissencephaly with midline calcification (Fig. 1). The combination of type I lissencephaly and midline calcification is reportedly pathognomonic of MDS [1]. MDS with 17p13.3 deletion in conventional G-band chromosome analysis and fluorescence in situ hybridization was diagnosed. She had an epileptic encephalopathy with infantile spasms (West syndrome) with a typical hypsarrhythmia pattern on her electroencephalogram (EEG). Echocardiogram had shown a mild patent ductus arteriosus and a mild aortic valve stenosis, which were medically treated. Repeated hospitalization had been required for aspiration pneumonia.

**Fig. 2:**
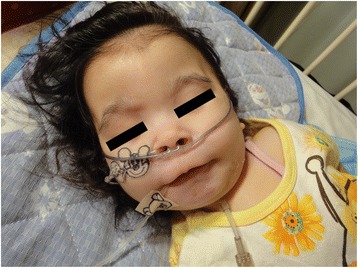
Facial photograph of the patient. The typical facial features of Miller–Dieker syndrome (MDS) are seen. These features include prominent forehead, bitemporal hollowing, short nose with upturned nares, prominent upper lip, and micrognathia

Pre-anesthetic examination of the child showed the typical facial features of MDS, including a prominent forehead, bitemporal hollowing, short nose with upturned nares, prominent upper lip, and micrognathia (Fig. 2). The pulse rate was 115 beats/min and blood pressure was 100⁄40 mmHg. The pulse oximetric oxygen saturation was 100 % while receiving oxygen 0.5 L/min through a nasal cannula. She had mild anemia with hemoglobin of 9.6 g/dL. Serum electrolytes, liver function tests, renal function tests, blood sugar, and electrocardiogram (ECG) were within normal limits. Chest X-ray showed no evidence of pneumonia or other abnormalities.

The patient was fasted for over 8 h with intravenous hydration to minimize the risk of aspiration during surgery. No premedication was given. Equipment for difficult intubation was prepared, and two anesthesiologists were present in the operation room. On arrival at the theater, pulse oximetry, ECG, and noninvasive blood pressure monitoring were established. Bispectral Index (BIS) electrodes (BIS Pediatric Sensor; Covidien, Minneapolis, MN, USA) were placed on the patient’s forehead and temples according to the manufacturer’s recommendations. Although BIS monitoring is primarily an objective means of assessing the level of sedation in adults, it is reported that BIS monitor is reliable for measuring depth of sedation in children aged more than 1 year [3]. The BIS value is a number between 0 and 100, BIS values near 100 being considered “awake”. Maintenance of a BIS value between 40 and 60 denotes adequate sedation during general anesthesia. However, the range of BIS values in this child was 16–21 before induction of general anesthesia. General anesthesia was induced with midazolam (0.3 mg/kg) and fentanyl (3 μg/kg). Rocuronium (1 mg/kg) was administered to facilitate tracheal intubation and to reduce time of mask ventilation. When an appropriate depth of anesthesia had been reached, laryngoscopy was performed using a Macintosh blade (size no. 2). The vocal cords were easily visualized, and a 4.0-mm uncuffed tracheal tube was inserted. No air leakage was detected at a pressure of 20 cm H_2_O. Anesthesia was maintained with sevoflurane 2.5 % and remifentanil 0.1–0.2 μg/kg/min with intermittent doses of rocuronium and fentanyl intravenously. The BIS values were in a range of 15–34 during general anesthesia. Neuromuscular function was monitored using a TOF-Watch SX (MSD, Tokyo, Japan). At the end of the procedure, residual neuromuscular block was antagonized with sugammadex (2 mg/kg) after confirming a train of four (TOF) count of 2. For postoperative analgesia, 100 mg of acetaminophen was given intravenously. After the child had returned to her preanesthetic level of consciousness, BIS values showed a range of 26–33. Her recovery and postoperative course were uneventful.

### Discussion

This case report draws attention to the anesthetic considerations in a child with MDS. Micrognathia can make airway management difficult; however, this patient was easily intubated. The BIS values before and after induction of general anesthesia were similar. Most patients with MDS have epileptic encephalopathies of infantile spasms (West syndrome) [4]. Congenital heart diseases are also frequently associated with MDS [1].

Careful airway management is necessary because of micrognathia and possible lung damage after repeated episodes of pneumonia. Appropriate difficult airway equipment should be prepared prior to the induction of general anesthesia. We avoided the use of succinylcholine, because it can cause hyperkalemia and malignant hyperthermia. Two case reports of patients with Walker–Warburg syndrome, another type of lissencephaly, have been published [5, 6]; both described difficulties in airway management in patients with lissencephaly.

The BIS values of our patient were 15–34 during the anesthetic course. Denman et al. observed the BIS values of ASA physical status I or II children and infants. They found that BIS values in awake and anesthetized children and infants were similar to those in adults [7]. Davidson et al. reported a similar study in 25 infants and 24 older children undergoing circumcision during a sevoflurane–nitrous oxide general anesthesia. They showed that BIS values are increased in older children when the end-tidal concentrations of sevoflurane were decreased from 0.9 to 0.7 %, 0.5 %. No such relationship was seen in infants [8]. A review showed that BIS values can be used to guide the administration of general anesthetic agents in children over 2 years of age. BIS values are less reliable, however, in infants under the age of 6 months [9]. Valkenburg et al. reported a range of BIS values of 14–22 in an awake 2-year-old boy with MDS [2]. The authors speculated that the low BIS values might be a consequence of the characteristic EEG abnormalities in patients with West syndrome. Intravenous anesthetics are not recommended for maintenance of general anesthesia in patients with MDS because of insufficiency of BIS monitoring.

Seizures occur in >90 % of children with type I lissencephaly. Approximately 80 % have infantile spasms; however, the EEG does not show typical hypsarrhythmia in some patients [4]. Because their central nervous system abnormalities include type I lissencephaly and marked dilatation of the ventricles, we believe that some children with MDS may have increased intracranial pressure. The goals of perioperative anesthesia management are to keep the intracranial pressure and cerebral blood flow within a normal range. Although we selected sevoflurane as general anesthetic agent in this patient, sevoflurane should be used with caution because of its potential to increase seizure activity and intracranial pressure. In terms of monitoring adequate cerebral blood flow, cerebral oximetry might provide more information than BIS values. Administration of potentially epileptogenic drugs to patients with MDS is contraindicated.

Because children with MDS are prone to aspiration, the risk of aspiration should be minimized by ensuring an adequate duration of fasting. Anesthesiologists should prevent perioperative pulmonary dysfunction and clear secretions in the airways. MDS may also be associated with congenital heart diseases, genitourinary abnormalities, and sacral dimples [1, 10].

Some children with MDS have dermal sinus tracts that communicate with the spinal canal [11]. The risk of spinal cord and/or nerve injury should be considered when caudal block or other regional anesthesia is planned in patients with MDS. Adequate maintenance of intravascular fluid volume and cardiac contractility is required in patients with severe congenital heart disease.

## Conclusions

In conclusion, we here report the anesthetic management of a child with MDS. The important anesthetic concerns in patients with MDS include potential difficult airways and extremely low BIS values.

## Consent

Written informed consent was obtained from the parent of the patient for the publication of this case report and any accompanying images. A copy of the written consent is available for review by the Editor-in-Chief of this journal.

## Abbreviations

BIS: Bispectral Index
ECG: electrocardiogram
EEG: electroencephalogram
MDS: Miller–Dieker syndrome
TOF: train of four
